# An Open Science MRI Database of over 100 Synaesthetic Brains and Accompanying Deep Phenotypic Information

**DOI:** 10.1038/s41597-023-02664-4

**Published:** 2023-11-04

**Authors:** Chris Racey, Christina Kampoureli, Oscar Bowen-Hill, Mathilde Bauer, Ivor Simpson, Charlotte Rae, Magda del Rio, Julia Simner, Jamie Ward

**Affiliations:** 1 School of Psychology and Sussex Neuroscience, University of Sussex, Brighton, UK; 2 School of Engineering and Informatics, University of Sussex, Brighton, UK

**Keywords:** Cognitive neuroscience, Human behaviour

## Abstract

We provide a neuroimaging database consisting of 102 synaesthetic brains using state-of-the-art 3 T MRI protocols from the Human Connectome Project (HCP) which is freely available to researchers. This database consists of structural (T1- and T2-weighted) images together with approximately 24 minutes of resting state data per participant. These protocols are designed to be inter-operable and reproducible so that others can add to the dataset or directly compare it against other normative or special samples. In addition, we provide a ‘deep phenotype’ of our sample which includes detailed information about each participant’s synaesthesia together with associated clinical and cognitive measures. This behavioural dataset, which also includes data from (N = 109) non-synaesthetes, is of importance in its own right and is openly available.

## Background & Summary

People with synaesthesia have remarkable ‘extra’ experiences of the world: words may have tastes, music may be seen (as well as heard), and numbers may form a spatial landscape. These atypical experiences, whilst of intrinsic interest, are also important because they are indicative of the presence of a neurodiverse phenotype that extends beyond synaesthesia itself. Synaesthetes perform differently on measures of cognition (often outperforming others^1^), they have a different style of thinking (e.g., more likely to be ‘visualizers’^2^), make different lifestyle choices^3^) and also possess a particular pattern of clinical vulnerabilities (more likely to have autism^4^ or develop PTSD following trauma^5^). One way of accounting for these seemingly different traits is to view synaesthesia as a product of an atypical neurodevelopmental cascade: from genetic differences; to atypical brain structure and function; to differences in cognition and behaviour of which the presence of synaesthesia itself is but one outcome^6^. When framed in this way, the study of synaesthesia becomes a paradigmatic case of individual differences in cognition and brain function, this being a current topic of widespread interest^7,8^.

Although there are many neuroimaging studies of synaesthesia^9^, there is a lack of consensus on the neural correlates of the condition^10^. The reported discrepancies likely stem from differences in imaging methodologies (VBM, DTI, fMRI), different characteristics of the synaesthetes themselves (which are not always documented in detail), and failures to replicate due to sample size inadequacy (N’s ~20 being typical). Furthermore, prior neuroimaging data on synaesthesia is not openly available.

We perform multimodal imaging based on international standards set by the Human Connectome Project, HCP^11,12^. The core aspect of the HCP approach is the division of the brain into distinct areas (termed parcellation) such that each individual cortical area can be differentiated from adjacent areas on at least one of several dimensions such as their pattern of resting state BOLD connectivity to other regions, or structural features such as cortical folding, myelination and cortical thickness^13^. No previous neuroimaging study of synaesthesia has approached it in this way despite neurodevelopmental differences in the formation of areal boundaries being a hypothesised key mechanism^14^ (parcellations derived from mapping to a standard atlas has been done^15^). Compared to voxel-based analyses, the HCP approach results in better spatial fidelity, increased statistical sensitivity and power, as well as robustness of cross-study comparisons^11^. However, the shared dataset is amenable to a wide variety of analytical strategies, including at the voxel level, and is by no means limited to the HCP approach.

Table 1 contains a summary of our dataset which consists of both neuroimaging data and also deep phenotypic information including the types of synaesthesia together with associated clinical and cognitive measures. The HCP Development and Ageing (HCP D/A) protocols^16^ were used so as to be inter-operable with other openly available datasets from normative and special populations (see the Usage Notes below), although we additionally scanned a local normative sample to enable comparisons across scanning centres.

**Table 1:** A summary of the neuroimaging and behavioral data including participants numbers (syn = synaesthetes; non-syn = non-synaesthetes).

Data type	Details	Participants
MRI T1-weighted image	Multiecho T1w MPRAGE (0.8 mm resolution; 8 mins 22 seconds)^49,50^	N = 102 syns N = 25 non-syns
MRI T2-weighted image	T2w SPACE (0.8 mm resolution; 6 mins 35 seconds)^49,50^	N = 102 syns N = 25 non-syns
fMRI Resting state data	BOLD resting state (four runs alternating AP and PA; 2 mm resolution, 488 volumes; each run lasted 6 mins 41 seconds)^49^	N = 102 syns N = 25 non-syns
HCP derived brain parcellations	Subject-specific parcellations (MSMAll), subject-specific node timeseries, and subject-specific parcellated connectomes^50^	N = 102 syns N = 25 non-syns
Behavioral data	Types of synaesthesia reported and consistency scores; questionnaire measures of: autistic traits, sensory sensitivity, anxiety, depression, stress, impact of events (related to PTSD), hypermobility, personality, and mental imagery; cognitive measures of: episodic memory for words, creativity (alternate uses), and intelligence (Raven’s matrices)^17^	N = 128 syns N = 109 non-syns

## Methods

The project was approved by the BSMS (Brighton and Sussex Medical School) Research Governance and Ethics Committee (reference ER/JAMIEW/23). Participants gave informed consent for both participation and data sharing. The procedure for data collection and preprocessing was pre-registered^17^ (https://osf.io/ycqgd/).

### Participants

A total of 237 participants completed all of the behavioural assessments. This included 128 people with synaesthesia (mean age = 36.01, S.D. = 13.42; gender 96:25:4 for female:male:non-binary) and 109 participants classed as not having synaesthesia (mean age = 34.59, S.D. = 13.11; gender 78:29:2 for female:male:non-binary). Neither age nor gender differed significantly across groups (age: t(235) = 0.704, p = 0.482; gender: X^2^ (1) = 1.304, p = 0.254 discarding non-binary simply due to small expected counts). Although gender is balanced across groups, the high proportion of women across both groups reflects recruitment challenges. Synaesthesia does not differ between men and women in terms of prevalence or characteristics^18^. Within this wider sample, the neuroimaging session was completed by N = 102 synaesthetes (mean age = 35.38, S.D. = 13.20; gender 78:23:1 for female:male:other) and N = 25 non-synaesthetes (mean age = 35.08, S.D. = 14.14; gender 19:6:0 for female:male:other). Fewer non-synaesthetes were scanned due to the wider availability of normative neuroimaging data using the same or similar protocols.

For the behavioural data, an a priori power analysis was performed using G*Power based on two-tailed independent t-tests with alpha = 0.05. At a power of 0.8, an N of 100 (per group) is sufficient for detecting a Cohen’s d of 0.40 and above. For brain-based biomarkers these are hard to estimate a priori (due to their multivariate nature), but there is no evidence that sample sizes beyond 100 are linked to improved classification accuracy^19^. Instead, classification accuracy depends on factors such as the ability to measure the trait in the first place, and the sophistication of the neuroimaging and classification protocols. For control data, other neuroimaging datasets using this protocol can be accessed to optimize power (see Usage Notes).

### Recruitment and inclusion criteria

#### Inclusion and exclusion criteria

For inclusion, participants had to be aged between 18–70 years of age. Synaesthetes must have passed a test to verify the presence of a recognised form of synaesthesia. Ward and Simner^20^ used clustering methods to identify ten broad groupings of synaesthesia (each typically made up of several different types of synaesthesia). These are listed in Table 2 together with methods used for verifying them in the present study, which rely on synaesthetes having high test-retest consistency for their associations. We further required that at least N = 50 synaesthetes should report three or more (/10) because we hypothesised that these ‘multi-synaesthetes’ would be associated with larger effect sizes (both in terms of behavior and brain-based biomarkers). That is, we employed purposive sampling and note the limitation that the sample may not be representative of the general population of synaesthetes and that this sample may have different motives for participation relative to controls. Most control participants were excluded if they self-reported synaesthesia (typically after the first online session), although we were subsequently able to verify three participants as having synaesthesia and they were reassigned to the synaesthete group. Participants were excluded if they did not complete in full the behavioural assessment (but the data was retained if they completed one session).

**Table 2:** Our synaesthetes were classed as having between one and ten clusters of types of synaesthesia (based on^20^), and all synaesthetes were objectively verified for at least one of these.

Synaesthetic Cluster	Description and examples	How verified? (in the present study)
Language-colour	Letters and numbers trigger colours (grapheme-colour synaesthesia) and/or words (e.g., names, months) trigger colours	High consistency of colour choices in immediate test-retest (3 repetitions of same stimulus) with a cut-off score of <1.43 indicative of synaesthesia^54,55^. A small number of synaesthetes were tested using verbal colour descriptions over a longer test-retest interval^56^.
Sequence-space	Sequences such as numbers, days, months have a particular pattern in space (e.g., lines, circles)	High consistency of spatial choices (xy coordinates) in immediate test-retest (3 repetitions of same stimulus) with a cut-off score of <0.203 indicative of synaesthesia and a questionnaire score < = 19^57^.
Visualised sensations	Music or pain or smell or taste (etc.) trigger visual experiences of (e.g.) colour and shape	Not applicable (only self-reported)
Personification	Thinking about (e.g.) letters and numbers as having genders and/or personalities	Not applicable (only self-reported)
Hearing-motion	Experiencing sounds from silent moving objects	Not applicable (only self-reported)
Tickertape	Visualising speech as written words (whether coloured or not)	Not applicable (only self-reported)
Mirror-touch	Seeing someone else touched triggers tactile sensations on ones own body	7 or more reports of tactile experiences in response to 14 videos depicting touch to a human body^58^
Language-taste	Words (etc.) trigger experiences of taste (lexical-gustatory synaesthesia)	High-consistency of taste ratings (sour, sweet, etc.) based on 2 repetitions of a word with a score >26% indicative of synaesthesia^59^.
Other smell/taste experiences	Music or other non-linguistic stimuli triggering smells or tastes	Not applicable (only self-reported)
Language-touch	Words, numbers, etc. experience touch-like bodily sensations	Not applicable (only self-reported)

Note that finer cuts of the phenomenology are also possible (e.g., separating out music-colour and pain-colour synaesthesias which, here, are grouped under the synaesthesia cluster of Visualised Sensations; see^20^).

Additional exclusion criteria were put into place for the MRI session. Participants were excluded for having a known or diagnosed neurological condition (e.g., epilepsy, multiple sclerosis, dementia, stroke; but migraine was an exception) following the HCP criteria^12^. Similarly, our exclusion criteria follow general guidance on MRI safety and tolerance also included in the HCP protocols (not pregnant, no unsafe metal, no moderate to severe claustrophobia). We did not exclude on the basis of diagnosed neurodevelopmental disorders (including autism) given our scientific interest in clinical vulnerabilities and co-morbidities in synaesthesia. Instead, our behavioural studies further ask about anxiety, stress, depression, and autistic traits so that other researchers can choose whether to exclude, include, or statistically explore any heterogeneity.

#### Recruitment of non-synaesthetes

Control participants were recruited from two sources. Firstly, from the University of Sussex community and other local participants (e.g., acquaintances). Secondly, from the recruitment website Prolific.

#### Recruitment of synaesthetes

**Fig. 1:**
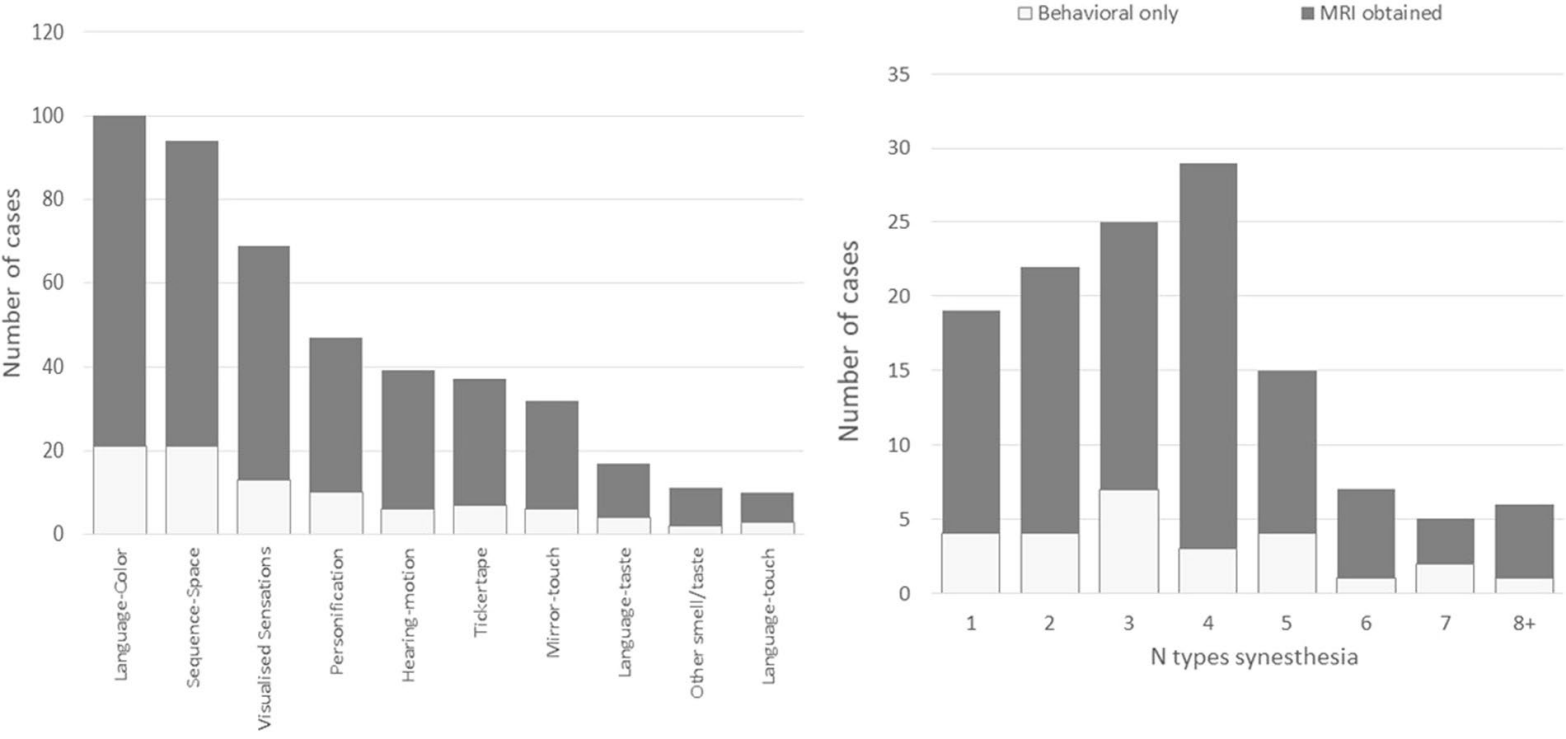
Top: The prevalence of different types of synaesthesia amongst the sample in this dataset (noting that the bars do not sum to the sample size, N = 128, because most synaesthetes report multiple types). Bottom: The distribution of the number of types of synaesthesia amongst the sample in this dataset (total N = 128).

Our synaesthete sample initially made contact via our website (www.sussex.ac.uk/synaesthesia) and filled in a questionnaire that asked about the types of synaesthesia they experienced. Synaesthetic experiences are defined in terms of an inducer (the type of stimulus that triggers it) and a concurrent (the type of response). For example, grapheme-colour synaesthesia is induced by graphemes (letters or numbers) and gives rise to a synaesthetic experience of colours. Most participants (since 2007) filled in an online version of this questionnaire in which inducers and concurrents were arranged as a grid of checkboxes (inducers in rows, concurrents in columns). An earlier downloadable paper-based version had participants draw a line between their relevant inducers and concurrents printed as two separate lists (both in columns). The full list of concurrents were: colours, shapes, taste, smell, noise, music, pain, and touch (an ‘other’ option was also included but is not considered further). The full list of inducers were: letters of the alphabet, English words, foreign words, peoples names, numbers, days of the week, months of the year, voices, pain, touch, body postures, music, noise, smell, taste, colour, shape and emotion. In both online and paper versions of the questionnaire, separate questions asked about the presence of sequence-space synaesthesia (e.g., spatial forms triggered by numbers) and sequence-personification synaesthesia (e.g., gender/personalities triggered by numbers). Since 2007 (i.e. the online version), we additionally asked about three other types of synaesthesia: tickertape (visualising speech as written words), hearing-motion (perceiving sound from silent moving objects), and mirror-touch synaesthesia (feeling touch on the body when watching another person being touched). These questions involved presenting participants with an image to illustrate the phenomenon (tickertape) or videos to induce the phenomenon (hearing-motion, mirror-touch). These slightly different screening questionnaires are shared in the online material together with both the raw responses and their recoding into the ten higher groupings (and the relevant scores used to verify the synaesthete). Figure 1 contains a summary of the types of synaesthesia that were reported and the distribution of number of types.

### Data acquisition and protocol overview

Synaesthetes had completed a previous diagnostic test to verify their synaesthesia and to report the nature of their experiences prior to enrolment, as already discussed in detail (Table 2). On agreeing to take part, participants were assigned a unique anonymised ID and given links to two sets of studies hosted on Qualtrics. The first set asked about synaesthesia itself and a set of clinically-related surveys. The second set related to personality and cognition and consisted of a mixture of surveys and cognitive tests.

After completion of the online parts, participants were then invited to take part in the neuroimaging session which lasted approximately 45 minutes. This consisted of the three imaging modalities that are essential to construct the HCP parcellation, namely resting state fMRI, T1-weighted structural MRI, and T2-weighted structural MRI. Data collection and analysis followed standardised protocols and pipelines to ensure that the data would be inter-operable with other datasets. The raw and preprocessed data are shared in the HCP data format. Additionally, we share the raw data in BIDS, Brain Imaging Data Structure, format^21^ ready for analysis in fmriprep^22^ or other neuroimaging processing pipelines.

### Behavioral measures

At the start of the online tests, participants entered their unique ID and also completed demographic questions relating to age, gender, handedness for writing (left, right) and highest education level (coded as 1 = up to 16, 2 = up to 18, 3 = undergraduate, 4 = postgraduate).

#### Assessment of synaesthesia

These were included (a) to determine whether controls report any types of synaesthesia (in which case we excluded or, in a few cases, verified them) and (b) to obtain further information about our synaesthetes including their projector-associator status (see below) where relevant, (c) to fill-in any missing data (e.g., not everyone had been previously asked about mirror-touch or hearing-motion), and (d) to ascertain the stability of their self-reported experiences. The questions were as follows (figures to illustrate questions 1–4 can be found in^17^):

1. “Do you experience colours for letters and numbers? (grapheme-colour synaesthesia)” YES/NO
  1a) If YES, participants are asked to complete the Projector-Associator questionnaire with N = 12 items^23^. Projectors experience their colours as if they were externally projected into space whereas Associators report colours in their mind’s eye or claim to know but not see colours.
2. “Some people always experience sequences in a particular spatial arrangement such as in these examples: [Figure]. Do you think that this applies to you?” YES/NO
  2b) If YES (2a), “Which of the following sequences do you visualise in this way? (tick all that apply) Numbers, Days, Months, Years, Letters of the Alphabet, Temperature, Height, Weight, Other [specify ____]”
  2c) If YES (2a), “Where are these sequences located? (choose one) In the space outside my body/Inside my body/On a screen that has no physical location”
3. “For some people, music and other kinds of sounds (e.g., voices, noises) trigger visual experiences such as coloured moving shapes. These are not like visual memories, but are more like abstract art. Do you think that this applies to you?” YES/NO
  3b) If YES, “Please could you give some examples or descriptions of what you experience….” [free text box]
4. “Some people always think of certain things (e.g., numbers) as having a gender (e.g., 5 is male) or a personality (e.g., 6 is bossy). Do you think this applies to you?” YES/NO
  4b) If YES, “Please could you give some examples of this….” [free text box]
5. “Did you feel touch on your face in response to seeing this? [https://youtu.be/aoUdvuLrawE]”. YES/NO
  5b) If YES (5a), “Where do you experience this?” [choose one] Left side/ right side/Not sure
  5c) If YES (5a), “Before taking this survey, had you ever noticed that you can feel touch just by seeing it in other people?” YES/NO
6. “The moving dots are silent, but some people hear something when they see the dots move. Did you hear something? [https://youtu.be/o39TiACe4mw]”. YES/NO
  6b) If YES (6a), “What did it sound like?” [free text]
  6c) If YES (6a), “Before taking this survey, had you ever noticed that you have auditory experiences to silently moving objects (that other people can’t hear)?”. YES/NO
7. “People with synaesthesia have unusual sensory experiences such as words triggering tastes, or pain trigging flashes of colour, or any other sensory combination. Aside from the answers above, do you think you have other forms of synaesthesia? If so, feel free to describe them here or to clarify any of your answers above.” [optional free text]

For controls to be classed as a potential synaesthete, and hence be excluded, we would need to observe an initial YES response followed by either a second confirmatory answer or a plausible example in the follow-up questions(s).

#### Clinical measures

For the clinical measures, we used the Autism Spectrum Quotient (AQ), the Glasgow Sensory Questionnaire (GSQ), the Anxiety Sensitivity Index (ASI-3), the Depression Anxiety Stress Scales (DASS) and the Impact of Events Scale-Revised (IES-R), with the latter related to PTSD (post-traumatic stress symptomatology). This was motivated by previous findings linking many of these conditions/traits to synaesthesia^5,24,25^. A further clinical measure - general joint hypermobility - was included as it has been linked to neurodiversity^26^ although there is no published data on synaesthesia.

##### Autism spectrum quotient (AQ)

The AQ is 50-item quotient including five subscales: attention switching, attention to detail, communication, imagination, social skill. The scores on each subscale range from 1–10^27^. Example items include “I would rather go to a library than a party” and “I enjoy doing things spontaneously.” Responses are given on a four-point scale (Definitely disagree, Slightly disagree, Slightly agree, Definitely agree) and recoded as 1 or 0 depending on whether the trait resembles an autistic behaviour or not.

##### Glasgow sensory questionnaire (GSQ)

The GSQ is a 42-item questionnaire and the total score ranges from 0–168^28^. Example items (for tactile sensitivity) include “Do you cut the labels out of your clothes?” and “Do you hate the feel or texture of certain foods in your mouth?” with responses given on a five-point scale coded from 0–4 (never, rarely, sometimes, often, always).

##### Anxiety sensitivity index – 3 (ASI-3)

The ASI-3 includes 18 questions that are divided in 3 subscales: cognitive, physical, social^29^. Example items include “I worry that other people will notice my anxiety” and “When I have trouble thinking clearly, I worry that there is something wrong with me.”. Responses are given on a five-point scale from 0–4 (very little, a little, some, much, very much). The scores on each subscale can range from 0–24

##### Depression, anxiety and stress scales (DASS)

The DASS is a 21-item questionnaire that includes three subscales: depression, anxiety, stress^30^. Participants are asked to reflect how much a statement applied to them over the last week and example items include “I couldn’t seem to experience any positive feeling at all” and “I found it difficult to relax”. Responses are given on a four-point scale from 0–3 (never, sometimes, often, almost always) and the scores for each subscale can range from 0–21.

##### Impact of event scale – revised (IES-R, linked to PTSD)

The IES-R is 22-item questionnaire with three subscales: avoidance (8 items, scores: 0–32), intrusion (8 items, scores: 0–32) and hyper-arousal (6 items, scores: 0–24)^31^. Participants were instructed to “please think about a significant stressful event from your life [highlights included in original]. This could be from any period of your life. Examples might include a bereavement, separation, injury, illness or any other event that was both significant and stressful for you.” They were then asked to state when this occurred. The date is included to make sure the participant focussed on a specific event/period in time. For the questions themselves they were asked to “indicate how distressing each difficulty has been for you during the past seven days with respect to this event. How much were you distressed or bothered by these difficulties? [highlights included in original].” Example items included “Pictures about it popped into my mind” and “I tried not to think about it.” Responses are given on a five-point scale from 0–4 (Not at all, Little bit, Moderately, Quite a bit, Extremely).

##### General joint hypermobility

Hypermobility was assessed using the five-part questionnaire, 5PQ^32^. This contains 5 questions with a yes/no response and examples include: “Can you now (or could you ever) bend your thumb to touch your forearm?” and “As a child did you amuse your friends by contorting your body into strange shapes OR could you do the splits?”. Participants are given a score between 0–5 (with a score of 2 or more being clinically indicative).

#### Cognitive and personality measures

For the cognitive and personality measures, we used the Big Five Inventory-2 (BFI-2), a Memory test, the Plymouth Sensory Imagery Questionnaire (PSI-Q) and the matrices reasoning test. This was motivated by previous findings linking these to synaesthesia^1,33,34^.

##### Big five inventory – 2 (Personality)

The BFI-2 includes 5 subscales: Extraversion, Agreeableness, Conscientiousness, Negative Emotionality, and Openness to Experience and the score on each subscale can range from 12–60^35^. Answers are framed with respect to “I am someone who…” and example items include “tends to be disorganized” and “is outgoing, sociable” with responses given on a 1–5 five-point scale (Disagree strongly, Disagree a little, Neutral – no opinion, Agree a little; Agree strongly).

##### Memory (learning phase)

For the study phase of the memory test, participants were firstly presented with 24 words and had to decide whether each word contained the letter “E” or not^36^. Words were presented for two seconds and participants were not told that they needed remember them. Instead, they were told it was a “Language test” to discourage use of a mnemonic strategy.

##### Creativity (Alternate Uses Test)

For the Alternate Uses Test adapted from^37^, participants were given a fixed time of 90 seconds (with a visible time counter) for each of six items (brick, car tyre, barrel, pencil, shoe, metal coat hanger). They were given the following set of introductory instructions: “You will be asked to produce as many different uses as you can think of, which are different from the normal use, for a number of common objects. For example…NEWSPAPER (typical use = for reading). Alternate use 1: swatting flies; Alternate use 2: line drawers; Alternate use 3: make a paper hat; Alternate use 4: writing a kidnap note; Alternate use 5: etc. You will be given points for the amount of different uses you generate. For example, ‘swatting flies’ and ‘swatting spiders’ aren’t sufficiently different uses, so the second one wouldn’t count. You will also get points for originality: for instance, ‘writing a kidnap note’ is quite a creative answer!”. For each item they were reminded to “Produce as many different uses as you can think of, which are different from the normal use, for a number of common objects. Remember your answers need to be different to each other and be as creative as possible”.

##### Memory (test phase)

The next block was labelled as a “Memory Test” and participants were instructed: “At the start of the study you were shown a series of words and had to decide if it contains an E or not. We will now test your memory for these words! Don’t worry if you find this hard - feel free to guess and just do your best.” Participants were then presented with 48 words (24 old, 24 new), one at a time, and asked to determine if they had seen it before (yes/no) and their degree of confidence on a four point scale (very confident, fairly confident, some confidence, guess)^36^. They had unlimited time to respond.

##### Mental imagery

The PSI-Q includes seven subscales: bodily sensations, emotions, sound, taste, touch, visual, smell and for each subscale the score ranges from 0 to 50^38^. Example items include “imagine the appearance of… a sunset” and “imagine the taste of… lemon” with responses given on an eleven-point scale (0 = no image at all; 10 = as vivid as real life).

##### Intelligence (Matrix Reasoning)

For the matrix reasoning test, the stimuli consist of 3 × 3 arrays of geometric shapes with one of the nine parts of the array missing^39^. Participants had to determine what should be in the missing part based on a forced choice set of six answers (labelled A-F). Participants were told that they had up to 20 minutes to complete the task (at which point it timed out), although they could complete it sooner if they wished. Participants were not able to go back and change or check a response after accepting it. There were eleven matrices so the scores could range from 0–11.

### MRI hardware and image acquisition

Using multimodal MRI scans acquired using HCP protocols (see below), it is possible to divide the brain into 180 parcellated areas in each hemisphere using a fully automatic processing pipeline. The parcellations are derived from a combination of T1-weighted (TIw), T2-weighted (T2w), and resting-state functional MRI (rfMRI)^13^. Due to time/financial constraints these were the only imaging sequences acquired. The more recent, and briefer, Development/Aging (D/A) HCP protocol was adopted, and the sequence was obtained from the Connectome Coordination Facility^16^. The scanner hardware at CISC mirrors exactly that being used at the imaging centres currently acquiring the HCP D/A dataset. It consists of a Siemens 3T Prisma scanner with a 32-channel head coil with multiband echo-planar imaging, EPI, sequences^16^.

The total scanning time for the sequences used are around 40 minutes. The field-of-view for all scans is positioned automatically using Siemens’ AutoAlign feature. It consists of the following sequences run in this order.

i) Spin echo field maps (AP, PA; 2 mm resolution; 18 seconds)
ii) BOLD resting state (runs 1 and 2 AP and PA; 2 mm resolution, 488 volumes; 2 × 6 mins 41 s; gradient-recalled echo, GRE, EPI sequence MB8 with TR/TE = 800/37 ms, flip angle = 52°; participant viewed white fixation cross on black background)
iii) Multiecho T1w MPRAGE (0.8 mm resolution; 8 mins 22 seconds; multi-echo TE = 1.8/3.6/5.4/7.2 ms with 6/8 slice partial Fourier; volumetric navigators, vNav, for prospective head-motion correction; participant viewed a TED talk of their choice)
iv) T2w SPACE (0.8 mm resolution; 6 mins 35 seconds; vNav correction; participant viewed a TED talk of their choice)
v) Spin echo field maps (AP, PA; 2 mm resolution; 18 seconds)
vi) BOLD resting state (runs 3 and 4 AP and PA; imaging parameters as described in ii)

### MRI Data Pre-processing

#### Data pre-processing and cortical parcellation

The HCP data pre-processing was carried out using FSL (FMRIB Software Library^40^;), FreeSurfer^41^, and the Connectome Workbench software^42^. The data preprocessing procedures followed the HCP pipelines (v4.3.0). PreFreeSurfer, FreeSurfer, PostFreeSurfer scripted workflows were used to process structural images. Gradient nonlinearity distortion correction, bias-field correction, high-pass spatial filtering, nonlinear normalization to 2 mm Montreal Neurological Institute (MNI) space, and segmentation/parcellation was applied. Structural surface registration used the Multimodal Surface Matching algorithm (MSMSulc). Subcortical and intracranial volumes were extracted from this using FreeSurfer v6.0 processing. This yielded 360 cortical parcels defined by the Glasser Multi-Modal parcellation (HCP-MMP^13^), as well as 19 subcortical volumetric regions estimated by MSMAll and other volumetric data computed by Freesurfer directly^43^.

Resting state fMRI data was processed using fMRIVolume and fMRISurface scripted pipelines. Preprocessing steps include gradient nonlinearity distortion correction, frame-wise rigid body registration to the single-band reference image to correct for head motion, dual-phase encoded spin-echo distortion correction, T1w-alignment, 2 mm MNI spatial normalization, and BOLD signal intensity normalization. Data was transformed into grayordinate space (registered to 32k_fs_LR mesh). Grayordinate space data are smoothed (2 mm FWHM; in volume-space subcortically and on the mesh cortical surface) and subjected to a high-pass filter of 0.005 Hz.

Time-series data from the four resting state scans was concatenated. Between-scan motion may impact data quality when combining different runs into a single analysis. FSL’s FLIRT^44^ with 12 degrees of freedom is used to estimate within-scan motion parameters between runs (X/Y/Z translation, pitch/roll/yaw and temporal derivatives); Mcflirt was used to estimate motion between consecutive volumes.

The final stages of functional processing are the ICA + FIX artefact removal procedure and MSMAll for surface-based functional alignment. Motion related, and other structured artefacts are removed by ICA + FIX processing (Independent Component Analysis followed by FMRIB’s ICA-based X-noiseifier^45,46^;). The FMRIB ICA + FIX preprocessing pipeline has been widely used in resting state fMRI studies. The MSMAll procedure uses T1w/T2w myelin maps and resting state network maps to align a subject’s cortical data to a group template.

#### Data quality control (QC)

All acquired data were subjected to the HCP standardised guidelines on quality control assessment including procedures for ensuring standardization in the acquisition of all measures across research assistants and across participants^42^. HCP data releases provide “qc_issue” codes for each participant to describe all assessed aspects of data quality. Participants are not excluded on this basis but data quality issues are recorded and reported to allow investigators to determine exclusion criteria at the analysis stage. The present study followed these procedures to assign ratings and qc_issue codes to all participants to ensure comparability with other HCP data releases. Raters were blind to group membership. The five categories of QC issue assessed were: anatomical anomalies, segmentation and surface QC, acquisition/coil related issues, artefacts in parcellation, manual reclassification of ICA-FIX components. Each of these were assessed by visually inspecting the data, and rating on a four point scale (1 = poor, 2 = fair, 3 = good, 4 = excellent). Anatomical scans are assessed for image crispness, clarity of the white and grey matter borders, to check for possible banding caused by subject motion and any other artifacts that could affect data quality. Automatically generated QC scenes for connectome workbench are used to assess freesurfer segmentation quality (accuracy of white/pial surfaces, segmentation errors, fused sulci or missing gyri), and inspect ICA + FIX components and their classification of signal/noise. The concatenated, cleaned timeseries data are provided with all noise components removed. Head movement during resting state was calculated as mean framewise displacement^47^.

## Data Records

Participants were assigned an anonymised code on enrolling for the study and were assigned a different code by the imaging centre (CISC) for the MRI data. An Excel spreadsheet containing the behavioural data and synaesthetic types, also lists both of these identifiers. This enables the synaesthetic phenotype, behavioural data, and MRI data to be linked.

The anatomical MRI images were anonymised by using the automated defacing software pydeface 2.0.2^48^. We also ensured removal of other potentially identifiable data (e.g., date of birth).

The MRI dataset consists of:

1. All raw MRI data (structural and functional) converted into BIDS format^49^ (https://openneuro.org/datasets/ds004466)
2. Images processed through the HCP pipeline consist of Freesurfer surface reconstructions, rfMRI data (1952 timepoints), subject-specific parcellations (MSMAll), subject-specific node timeseries, and subject-specific parcellated connectomes. Raw data in HCP format are also included to allow re-running of HCP processing with future versions of the pipeline. These data are available for download via the NeuroImaging Tools and Resource Collaboratory, NITRC (https://www.nitrc.org/projects/syn_hcp/)^50^, noting that each participant has 37 GB of data.
3. Derived structural data including parcellated (N = 360 cortical regions) cortical thickness, surface area, and myelination are available at^17^ (https://osf.io/ycqgd/) alongside volumetric data (e.g., of subcortical regions) extracted by Freesurfer

The behavioural dataset is available at^17^ (https://osf.io/ycqgd/) and consists of:

1. Qualtrics scripts used for collecting the online survey data, and other stimuli used by those scripts (including image files)
2. Full anonymised responses to the initial synaesthesia questionnaire (filled in by our participants prior to enrolling on this study), noting that there are several versions of this (depending on the year in which they volunteered for research).
3. Raw data and summary scores for all behavioural measures (each participant is a separate row in an Excel spreadsheet)
4. Scripts (in R and Matlab) for calculating the summary scores for the memory data
5. Scores from two (blind) independent raters for the creativity responses

## Technical Validation

### MRI data quality assessment

Our research yielded two incidental findings noted by the radiographers: a Chiari 1 malformation in one synaesthete and a small atypical mass outside the brain in another. These resulted in satisfactory quality control checks (e.g., for tissue segmentation) and were retained but are recorded in the dataset.

**Fig. 2:**
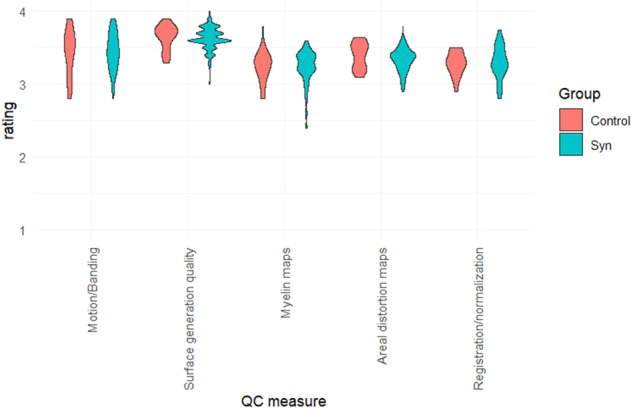
Quality control (QC) ratings for five structural imaging measures. Note: 4 = Excellent, 3 = Good, 2 = Fair, 1 = Poor.

The results of the structural QC checks are summarised in Fig. 2 with the majority falling in the good-to-excellent range (rated 3–4). There were no group differences in any of the structural QC measures: motion/banding (t(125) = 0.243, p = 0.809), Freesurfer surface generation quality t(125) = −0.429, p = 0.669), myelin maps (t(125) = 0.092, p = 0.92), areal distortion maps (t(125) = −1.605, p = 0.111), and registration/normalization to standard space t(125) = 0.351, p = 0.726).

**Fig. 3:**
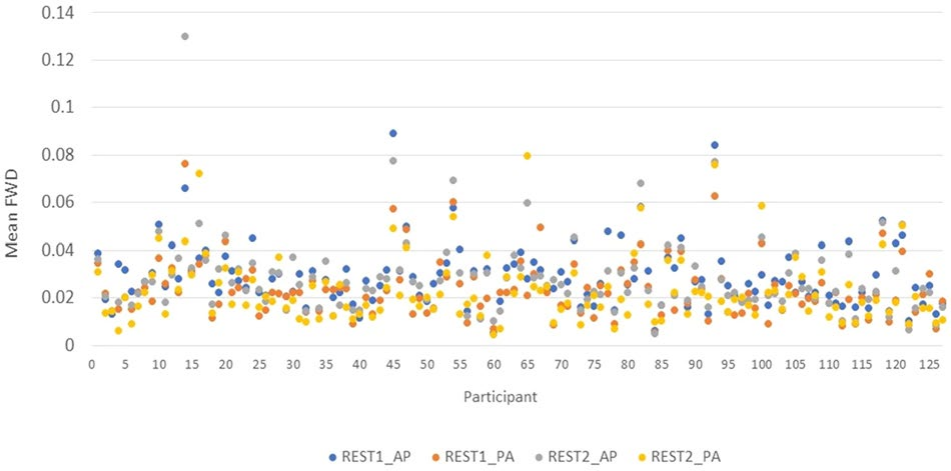
Mean framewise displacement (FWD) in cm for N = 127 participants (x-axis). The first N = 25 participants are non-synaesthetes. Data points are shown for each of four runs where runs were organised into blocks (REST 1 and REST2) acquired either anterior-to-posterior (AP) or posterior-to-anterior (PA).

To take head movement into account, the mean framewise displacement^47^ was calculated for each participant and each resting state run. The results are summarised in Fig. 3. There was no significant group difference in this measure (synaesthetes mean = 0.025, s.d. = 0.012; controls mean = 0.030, s.d. = 0.014; t(125) = −1.638, p = 0.102). Overall head movement was low and comparable to prior research. But there were some participants where one or more run had more substantial movement (FWD > 0.05 cm) that require consideration before use (e.g., matching across groups, deleting runs or participants).

### Behavioral measures quality assessment

Survey scores (the sum of responses) were computed for the questionnaires taking into account reverse coding and separate subscale scores as appropriate. Cronbach’s alpha serves as a measure of reliability (i.e., the extent to which different items reflect the same underlying construct), and these are listed in Table 3. The lowest two values (AQ social skills, and 5PQ hypermobility) had ‘questionable’ reliability but such values are common for the AQ^51^. Cronbach’s alpha is not previously reported for the 5PQ but has shown to be reliable in other ways (test-retest scores).

Scoring of the creativity measure (AUT) is normally done by human judgment. Here we use two different metrics that have been shown to have good external validity in terms of their degree of association to cognitive measures related to creativity (working memory, intelligence) and in terms of tracking differences in brain structure and brain activity^52,53^. At the level of individual responses, two raters judged the novelty of the response (0 = *extremely common*, 5 = *extremely uncommon*). At the level of items (e.g., brick), the two raters were presented with the set of responses from a given individual and asked to rate the overall creativity (0 = *not at all creative*, 5 = *extremely creative*). An initial set of 50 sets of responses (out of 237 × 6 = 1,422) acted as a calibration set where discrepancies between raters were discussed and, following this, the entire set was scored again independently. Both raters were psychology researchers and were given randomised data (separately for the different ratings) fully blinded to group membership or any other participant detail. For judgments of creativity, the inter-rater reliability for the six objects were r = 0.42 (barrel), 0.51 (pencil), 0.53 (car tyre), 0.56 (brick), 0.61 (shoe) and 0.61 (metal coathanger), all p < 0.001. When averaged across the six objects (i.e. a single value for each participant from each rater), the inter-rater reliability for creativity was r = 0.81. For judgments of novelty, the inter-rater reliability by object were were r = 0.27 (barrel), 0.54 (car tyre), 0.56 (brick), 0.59 (pencil), 0.64 (metal coathanger) and 0.68 (shoe), all p < 0.001. The inter-rater reliability, after averaging across the 6 objects, was r = 0.56. Deleting the scores for least reliable object (barrel) led to little or no improvement for overall reliability (r = 0.79 for creativity and r = 0.62 for novelty) so all data were retained.

**Table 3:** Cronbach’s alpha reliability scores for the various survey measures.

Measure	Overall scale	Subscales
Autism quotient (AQ)	α = 0.893	Attention switching: α = 0.798 Attention-to-detail: α = 0.773 Communication: α = 0.823 Imagination: α = 0.729 Social skills: α = 0.663
Glasgow Sensory Questionnaire (GSQ)	α = 0.936	NA
Anxiety Sensitivity Index (ASI-3)	α = 0.924	Cognitive: α = 0.897 Physical: α = 0.873 Social: α = 0.835
Depression, Anxiety, Stress Scale (DASS-21)	α = 0.937	Depression: α = 0.924 Anxiety: α = 0.833 Stress: α = 0.876
Impact of Events Scale (IES-R)	α = 0.958	Avoidance: α = 0.882 Hyperarousal: α = 0.898 Intrusion: α = 0.939
Hypermobility (5-PQ)	α = 0.671	NA
Big Five Inventory (BFI-2)	NA	Extraversion: α = 0.878 Agreeableness: α = 0.784 Conscientiousness: α = 0.868 Negative emotionality: α = 0.919 Openness to experience: α = 0.839
Plymouth Sensory Imagery Questionnaire (PSI-Q)	α = 0.964	Bodily sensations: α = 0.893 Emotions: α = 0.864 Sounds: α = 0.858 Taste: α = 0.917 Touch: α = 0.910 Visual: α = 0.867 Smell α = 0.927

By convention, α > 0.9 is ‘excellent’, 0.9 > α > 0.8 is ‘good’, 0.8 > α > 0.7 is ‘acceptable’, 0.7 > α > 0.6 is ‘questionable’, 0.6 > α > 0.5 is ‘poor’, and α < 0.5 is ‘unacceptable’. NA = not applicable.

## Usage Notes

Our neuroimaging dataset is openly available in two formats: a BIDS format and HCP format (see Data Records). The accompanying demographic and behavioral data is available for download at^17^ (https://osf.io/ycqgd/).

Other datasets from normative and special populations acquired using the same HCP D/A sequence and analysis scripts are available via the NIMH Data Archive (https://nda.nih.gov/), which requires an institutional license for data access and use. The HCP YA database (https://registry.opendata.aws/hcp-openaccess/) used older acquisition and analysis protocols and researchers are advised to check interoperability before use.

## Data Availability

The scanning sequence for HCP D/A is available on request from the Connectome Coordination Facility (https://nda.nih.gov/ccf/). The data processing used the openly accessible HCP pipeline version 4.3.0 (https://github.com/Washington-University/HCPpipelines/releases/tag/v4.3.0) and Freesurfer version 6.0 (https://surfer.nmr.mgh.harvard.edu/pub/dist/freesurfer/) run on the connectome workbench version 1.4.2 (https://www.humanconnectome.org/software/get-connectome-workbench).
